# Probiotics and the Human Microbiome: Classical Functions, Emerging Systemic Roles, and Future Therapeutic Frontiers

**DOI:** 10.3390/biology15090665

**Published:** 2026-04-23

**Authors:** Imen Zalila-Kolsi, Ray Al-Barazie

**Affiliations:** College of Medical and Health Sciences, Liwa University, Abu Dhabi P.O. Box 41009, United Arab Emirates; ray.albarazie@lu.ac.ae

**Keywords:** probiotics, microbiome, gut–brain axis, immune modulation, metabolic health, personalized therapy, synthetic biology

## Abstract

Probiotics, live microorganisms that confer health benefits when administered in adequate amounts, are increasingly recognized as modulators of interconnected microbiome–host networks that extend beyond gastrointestinal function. This review synthesizes evidence on classical probiotic roles in maintaining gut homeostasis, immune regulation, and infection prevention, while integrating emerging systemic effects across the gut–brain, gut–skin, gut–oral, and metabolic axes. Rather than presenting isolated outcomes, we adopt a systems-level framework that links probiotic actions to shared mechanisms, including microbial metabolite signaling (e.g., SCFAs), competitive exclusion of pathobionts, barrier reinforcement, and immune–neuroendocrine pathway modulation. We further discuss translational advances that enable rational probiotic design, including targeted delivery platforms (encapsulation and protective matrices), engineered/next-generation strains, and postbiotic-inspired strategies, alongside sustainability considerations and regulatory/labeling challenges. Finally, we outline future directions emphasizing precision microbiome-centered interventions, synthetic biology, and AI-assisted multi-omics analysis to support strain- and context-specific probiotic strategies. Collectively, this review provides an integrated, systems-oriented synthesis to guide future research and accelerate safe clinical and industrial applications of probiotics.

## Simple Summary

This article explains how probiotics, live microorganisms including beneficial bacteria and yeasts, can support overall health. They are known for improving digestion, but research shows they may also help with dental health, skin conditions, mental wellbeing, and the control of blood sugar, cholesterol, and body weight. Probiotics work by blocking harmful germs, producing natural protective substances, and supporting the body’s defenses. The article also introduces new ways to make probiotics more effective and discusses the need for better safety, quality, and regulation. Understanding how probiotics work may lead to safer and more personalized ways to improve everyday health.

## 1. Introduction

The term “probiotics,” which comes from the Greek word “for life,” refers to live microorganisms that, when given in sufficient quantities, promote the host’s health [1]. By regulating gut microbiota, boosting immune responses, and generating bioactive substances like bacteriocins and short-chain fatty acids (SCFAs), these advantageous microorganisms, mostly non-pathogenic bacteria like *Lactobacillus*, *Bifidobacterium*, *Enterococcus*, and *Streptococcus*, as well as clinically relevant yeast probiotics such as *Saccharomyces boulardii,* play a critical role in preserving host health [2,3,4,5].

Probiotics are not a novel idea. Their use can be traced back to ancient cultures like the Greeks and Romans, who drank fermented milk for its health benefits. Elie Metchnikoff’s hypothesis that fermented dairy products are linked to longevity marked the beginning of the scientific investigation into probiotics in the early 1900s [6]. Lilley and Stillwell originally used the term “probiotic” in 1965 to refer to compounds that support microbial development. Over time, the term’s meaning changed to highlight the survivability and health-promoting benefits of live microorganisms [7].

Research on the systemic effects of probiotics has extended beyond the gastrointestinal tract in recent decades. Growing knowledge of the human microbiome, a complex ecology of trillions of microbes living in the gut, skin, oral cavity, and respiratory tract, is a major factor in this change [4,8,9]. It is now understood that microbiomes play a crucial role in determining human health, impacting immune response, digestion, metabolism, and even cerebral function. An imbalance in microbial populations, known as dysbiosis, has been linked to a variety of illnesses, including cancer, neurological diseases, autoimmune diseases, and metabolic disorders [3,10,11,12].

Given the growing interest in probiotics and the intricate nature of microbiome science, this review emphasizes their traditional functions in promoting gut health and modulating the immune system, while also examining the emerging systemic impacts on mental wellbeing, skin health, oral hygiene, and metabolic processes. It elaborates on essential mechanisms of action, novel delivery methods, and current obstacles such as regulatory issues, safety concerns, and strain specificity. The topic of sustainability is also considered, especially regarding the creation of environmentally friendly probiotic production techniques, biodegradable delivery systems, and the utilization of food-based carriers to minimize pharmaceutical waste [12]. Ultimately, the review delineates prospective avenues for research, including personalized probiotics, advancements in synthetic biology, and AI-enhanced microbiome studies, underscoring the necessity for integrative and sustainable strategies in the field of probiotic science. This interdisciplinary review integrates microbiology, immunology, neuroscience, dermatology, and biotechnology to provide a comprehensive understanding of probiotics and their evolving role in human health. Probiotic research has evolved beyond traditional gastrointestinal applications, revealing complex microbiome-mediated interactions that influence multiple organ systems and physiological processes. This review adopts a systems-level perspective to synthesize emerging evidence on probiotic-driven gut–brain, gut–skin, gut–oral, and metabolic interactions, emphasizing shared mechanisms rather than isolated clinical outcomes. By integrating microbial metabolites, immune modulation, and host signaling networks, the review introduces a unified mechanistic framework that links systemic probiotic effects to therapeutic function. In addition, it advances a forward-looking translational perspective, spanning engineered probiotics, advanced delivery strategies, and AI-assisted microbiome analysis, that connects mechanistic insight with the rational design of next-generation probiotic interventions.

## 2. Classical Roles of Probiotics

### 2.1. Gut Health and Digestion

**Figure 1:**
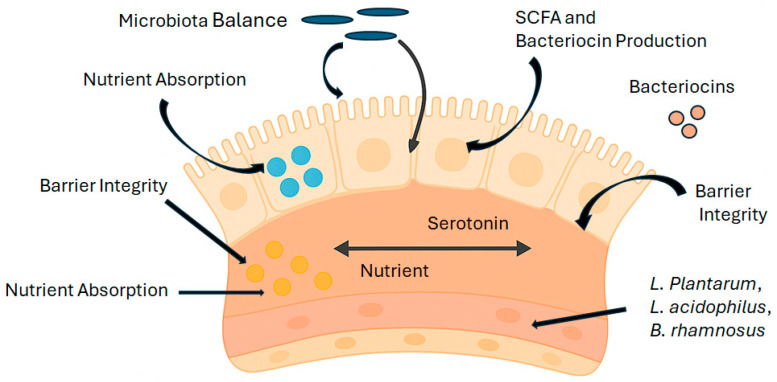
Mechanistic overview of probiotic contributions to gut health and digestive function.

Probiotics, especially those from the *Lactobacillus* and *Bifidobacterium* genera, have been acknowledged for their traditional role in fostering gut health and facilitating digestion. These advantageous microorganisms play a crucial role in sustaining a balanced gut microbiota, improving the integrity of the mucosal barrier, and aiding in nutrient absorption [13,14] (Figure 1).

Mechanistically, probiotics promote gastrointestinal tract (GIT) health through the production of antimicrobial metabolites, including bacteriocins and short-chain fatty acids (SCFAs), which suppress pathogenic bacteria and help regulate the intestinal microenvironment [15]. In parallel, advances in encapsulation and delivery technologies have improved probiotic survival during gastrointestinal transit, thereby enhancing their functional efficacy within the gut.

Functionally, experimental and clinical studies indicate that strain-specific *Lactobacillus* species, such as *L. plantarum*, *L. acidophilus*, and *L. rhamnosus*, regulate gut motility and carbohydrate metabolism, contributing to improved lactose digestion and alleviation of constipation and bloating [14]. These effects are partly mediated by probiotic-induced modulation of neurotransmitter signaling within the enteric nervous system, influencing colonic motility and digestive coordination. Furthermore, probiotic supplementation has been shown to reinforce intestinal barrier integrity by increasing tight junction protein expression and reducing epithelial permeability, as reflected by decreased serum zonulin levels and lower systemic inflammatory markers, including C-reactive protein (CRP) and TNF-α, in randomized controlled trials [16].

In clinical contexts, the digestive and barrier-protective effects of probiotics translate into more consistent therapeutic benefits in ulcerative colitis, whereas evidence supporting efficacy in Crohn’s disease remains limited and inconclusive. Early randomized controlled trials established proof of concept for probiotic efficacy in ulcerative colitis, including a double-blind, placebo-controlled study demonstrating that VSL#3 significantly reduced disease activity as adjunctive therapy in relapsing mild-to-moderate disease [17], as well as a large double-blind, double-dummy trial showing that *Escherichia coli* Nissle 1917 was equivalent to mesalazine in maintaining remission over 12 months [18]. More recent randomized double-blind, placebo-controlled trials have reinforced these findings, reporting significant reductions in intestinal inflammation and fecal calprotectin levels with multi-strain probiotic formulations in ulcerative colitis, while consistently failing to demonstrate comparable benefits in Crohn’s disease [19,20].

Collectively, these primary clinical studies, spanning early landmark trials and contemporary investigations, support the established, disease-specific role of selected probiotic formulations in ulcerative colitis through mechanisms involving immune modulation, restoration of microbial balance, and reinforcement of intestinal barrier integrity [21].

The key probiotic functions related to gut health and digestion are summarized in Table 1.

### 2.2. Immune System Modulation

Probiotics have been acknowledged for their capacity to regulate the immune system, a traditional function that has received growing scientific support [22]. These living microorganisms, especially those from the *Lactobacillus* and *Bifidobacterium* genera, engage with the immune cells of the host to improve both innate and adaptive immune responses. As noted by Mazziotta et al. (2023), probiotics affect immune homeostasis by interacting with dendritic cells, macrophages, and lymphocytes within the gut-associated lymphoid tissue (GALT), thus facilitating the activation of regulatory T cells (Treg) and the production of anti-inflammatory cytokines such as IL-10 and TGF-β [22]. This interaction is crucial for sustaining immune tolerance while bolstering the host’s defense against pathogens [22].

Recent research indicates that probiotics can enhance mucosal immunity by boosting the production of secretory IgA (sIgA), which is essential for neutralizing pathogens and preserving gut barrier integrity. A systematic review and meta-analysis conducted by Zheng et al. (2023) validated that probiotic supplementation significantly lowered inflammatory markers such as CRP, TNF-α, and IL-6, while also improving gut barrier function, which is intricately associated with immune regulation [16]. Moreover, Singh et al. (2023) highlighted that probiotics have the potential to restore immune balance in states of dysbiosis, consequently diminishing the risk of chronic inflammation and autoimmune disorders [23].

Strain-specific effects have been recorded as well. For example, *Lactobacillus acidophilus* and *Bifidobacterium animalis* subsp. *lactis* have shown synergistic anti-inflammatory effects in vitro by influencing NF-κB and MAPK signaling pathways, decreasing pro-inflammatory cytokine expression, and boosting TLR2-mediated immune responses. These results highlight the traditional immunomodulatory function of probiotics, which is crucial not only for sustaining immune balance but also for providing therapeutic possibilities in the treatment of inflammatory and autoimmune disorders [24].

Table 2 provides an overview of the main immunomodulatory roles and mechanisms associated with probiotic strains.

### 2.3. Prevention of Gastrointestinal Infections

Probiotics have shown considerable promise in preventing gastrointestinal (GI) infections through various mechanisms, such as the competitive exclusion of pathogens, enhancement of mucosal barrier function, and modulation of immune responses [25]. A thorough umbrella meta-analysis conducted by Zeng et al. (2025) revealed that probiotic supplementation significantly lowered the risk of diarrhea (RR 0.44), nausea, bloating, and epigastric pain across a range of GI conditions, including infections caused by *Helicobacter pylori* and *Clostridioides difficile* [26]. These effects were especially notable in studies utilizing multi-strain formulations and shorter intervention periods [26]. Likewise, a systematic review by Goodman et al. (2021) determined that the co-administration of probiotics with antibiotics decreased the occurrence of antibiotic-associated diarrhea (AAD) by 37%, with *Lactobacillus* and *Bifidobacterium* strains exhibiting the most reliable efficacy [27].

Probiotics exert their protective effects mechanistically by generating antimicrobial substances, including bacteriocins, organic acids, and hydrogen peroxide, which serve to inhibit the colonization of pathogens [28]. Zhu et al. (2023) emphasized the significance of *Bacillus* species in the production of structurally varied metabolites that specifically target enteric pathogens and bolster host immunity, thus alleviating the impact of drug-resistant infections [29]. These spore-forming probiotics also engage in competition for nutrients and adhesion sites, strengthen the integrity of the gut barrier, and modify the gut environment to promote beneficial microbes [29]. Additionally, Milner et al. (2021) conducted a review of both in vitro and in vivo studies that demonstrated how strains such as *Lactobacillus rhamnosus*, *Bifidobacterium lactis*, and *Saccharomyces boulardii* inhibit the proliferation of pathogens like *E. coli*, *Shigella*, and *C. difficile*, while simultaneously decreasing the production of pro-inflammatory cytokines [30]. Yeast probiotics have a unique and significant role in maintaining gastrointestinal health. *Saccharomyces boulardii* is a recognized probiotic yeast known for its effectiveness in preventing and treating antibiotic-associated diarrhea and recurrent *Clostridioides difficile* infection [31,32]. In contrast to bacterial probiotics, *S. boulardii* possesses inherent resistance to antibiotics and produces its positive effects via distinct mechanisms, such as neutralizing bacterial toxins, strengthening intestinal barrier function, and altering host immune reactions [33,34]. These characteristics enable yeast probiotics to enhance bacterial strains, especially during antibiotic treatment, by aiding microbiome stability and decreasing inflammation caused by pathogens.

These findings collectively highlight the traditional function of probiotics as a secure and effective supplementary measure in the prevention of gastrointestinal infections, especially among at-risk groups such as individuals receiving antibiotic treatment or those in a hospital setting. Their capacity to restore microbial equilibrium, bolster mucosal defenses, and diminish pathogen levels establishes them as a viable alternative or complement to standard antimicrobial approaches.

The principal probiotic mechanisms involved in preventing gastrointestinal infections are summarized in Table 3.

## 3. Emerging Systemic Roles

### 3.1. Neuroprobiotics and the Gut–Brain Axis

The gut–brain axis (GBA) constitutes a sophisticated bidirectional communication network linking the gastrointestinal tract with the central nervous system, facilitated by neural, endocrine, immune, and metabolic pathways. Recent studies have underscored the promise of neuroprobiotics, probiotic strains that offer neurological advantages, in modulating this axis and affecting mental health outcomes [35]. A systematic review conducted by Crocetta et al. (2024), employing neuroimaging techniques such as fMRI, demonstrated that probiotics can modify brain activity in areas related to emotional regulation and cognitive processing, including the amygdala, precuneus, and orbitofrontal cortex [36]. These effects are supported by experimental and clinical evidence indicating that probiotic-induced alterations in gut microbial composition influence central neural circuits via vagal nerve signaling and immune–neuroendocrine pathways [37,38]. In clinical populations, including those with major depressive disorder (MDD) and irritable bowel syndrome (IBS), probiotics have been found to normalize brain function and enhance connectivity within mood-regulating networks such as the subcallosal cortex and hippocampus [36].

Strain-specific effects have been documented as well. A meta-analysis conducted by Rahmannia et al. (2024) revealed that probiotics containing *Lactobacillus acidophilus*, *L. paracasei*, *L. plantarum*, and *Bifidobacterium bifidum* significantly alleviated depressive symptoms, especially when evaluated using the Beck Depression Inventory (BDI) [39]. These effects are believed to be facilitated by the production of neurotransmitters such as serotonin and gamma-aminobutyric acid (GABA), the modulation of the hypothalamic–pituitary–adrenal (HPA) axis, and a decrease in systemic inflammation [39,40,41].

In the realm of neurodegenerative disorders, probiotics have exhibited potential in influencing brain-derived neurotrophic factor (BDNF), which is a crucial regulator of neuroplasticity and cognitive abilities [42]. A meta-analysis conducted by Hashemi et al. (2025), encompassing 20 randomized controlled trials, revealed that probiotic supplementation notably elevated serum BDNF levels, particularly with interventions extending beyond 10 weeks [42]. This indicates a possible function for probiotics in promoting brain health and reducing cognitive deterioration. Moreover, Singh (2024) highlighted the significance of gut dysbiosis in worsening neurological conditions such as Alzheimer’s disease, Parkinson’s disease, and schizophrenia [43]. Dysbiosis can compromise the integrity of the gut barrier, permitting pro-inflammatory molecules to access the brain and initiate neuroinflammation. Probiotics may mitigate these impacts by restoring microbial equilibrium, boosting short-chain fatty acid production, and modulating immune responses [43].

In summary, these findings emphasize the growing systemic importance of probiotics in influencing the gut–brain axis, presenting promising opportunities for supplementary therapies in mood disorders, cognitive decline, and neurodegenerative diseases. Nevertheless, additional longitudinal and mechanistic research is required to elucidate strain-specific effects, optimal dosages, and long-term safety.

### 3.2. Influence on Mood, Anxiety, Depression, and Neurodegenerative Diseases

Recent studies have increasingly underscored the ability of probiotics to affect mental health via the gut–brain axis, especially in the regulation of mood, anxiety, depression, and neurodegenerative disorders [35,44,45]. A thorough meta-analysis conducted by Rahmannia et al. (2024) examined 12 randomized controlled trials (RCTs) with a total of 707 participants and discovered that probiotics containing strains such as *Lactobacillus acidophilus*, *L. paracasei*, *L. plantarum*, and *Bifidobacterium bifidum* significantly alleviated depressive symptoms as measured by the Beck Depression Inventory (BDI), yielding a mean difference of −2.69 (95% CI: −4.22 to −1.16; *p* < 0.001) [39]. Nevertheless, findings were inconsistent across other measures like HAMD and DASS, indicating that the specificity of strains and the tools used for assessment are vital in determining the effectiveness of probiotics [39].

In a recent study conducted by Leiden University, it was found that daily mood tracking indicated probiotics could alleviate negative emotions such as stress, anxiety, and fatigue within a mere two weeks of supplementation. Participants who consumed *Lactobacillus* and *Bifidobacterium* strains exhibited enhanced emotional regulation and a more acute perception of emotional cues when compared to those in placebo groups [46]. These results bolster the hypothesis that probiotics may affect mood through mechanisms related to the vagus nerve, immune modulation, and hormonal signaling. In addition to mood disorders, probiotics have demonstrated potential in the treatment of neurodegenerative diseases. A systematic review by Ojha et al. (2023) determined that strains such as *Lactobacillus acidophilus*, *L. casei*, and *Bifidobacterium bifidum* can influence inflammation and oxidative stress, critical factors in neurodegeneration, by restoring the balance of gut microbiota [47]. These effects may contribute to the prevention of cognitive decline and the maintenance of neuronal health [47].

All of these results highlight the potential of probiotics as supplemental treatments for neurodegenerative illnesses and mood disorders. However, issues with strain specificity, the best dosage, and long-term effectiveness continue to plague the field. Future studies should focus on individualized strategies, combining multi-omics analysis and microbiota characterization to customize probiotic treatments for specific patients.

### 3.3. Skin Microbiome and Dermatological Health

Our knowledge of the skin microbiome’s function in dermatological health has greatly expanded as a result of recent research that has shown how it affects both inflammatory and chronic skin disorders. Microbial dysbiosis, specifically the overgrowth of *Staphylococcus aureus* and decreased microbial diversity, is a common characteristic of disorders such as atopic dermatitis (AD), psoriasis, and rosacea, according to Lee et al.’s scoping analysis of 38 articles from 2025. Their research showed that whole-genome sequencing is more effective than 16S rRNA at detecting strain-level variations, which are essential for distinguishing between commensal and pathogenic functions [48]. Prajapati et al. (2025) investigated the new area of postbiotics, which are non-viable microbial cells or their metabolites, and showed how they might improve wound healing, lower inflammation, and boost skin immunity [49].

Interestingly, it was demonstrated that *Staphylococcus epidermidis* activates IL-17A+ CD8+ T cells, indicating a protective function in skin immunity. In their assessment of microbiome-based treatments, Rušanac et al. (2025) emphasized the potential of probiotics, prebiotics, and live biotherapeutics to suppress pathogenic colonization and restore microbial equilibrium [50]. As safer substitutes for traditional antibiotics and corticosteroids, their findings lend credence to the development of medicines that target the microbiota [50]. A fundamental summary of the biogeography of the skin microbiome was given by Whiting et al. (2024), who pointed out that various skin locations are home to unique microbial communities and that therapies like moisturizers and prebiotic compounds may aid in reestablishing microbial balance [51].

Lastly, Cymbiotics Biopharma’s evaluation from 2025 described condition-specific microbial alterations, including a drop in *Lactobacillus* in AD and an increase in Firmicutes in psoriasis, and suggested innovative delivery techniques such as ultrasound-enhanced metabolite administration [52]. Together, these investigations highlight the therapeutic potential of microbiome modification in dermatology and advocate for individualized, strain-specific strategies backed by strong clinical evidence.

### 3.4. Role in Acne, Eczema, and Wound Healing

Probiotics have demonstrated increasing therapeutic potential in the management of skin conditions such as acne, eczema, and wound healing by modulating cutaneous microbial communities and inflammatory responses [53,54]. A systematic review from 2024 by Boby et al. analyzed nine clinical trials evaluating oral and topical probiotics for acne vulgaris. The results showed that strains such as *Lactobacillus plantarum*, *L. rhamnosus* GG, and *Enterococcus faecalis* notably lowered acne lesion numbers, enhanced skin hydration, and adjusted the skin microbiome by reducing *Cutibacterium acnes* and *Staphylococcus aureus* levels. These impacts are linked to the anti-inflammatory characteristics of probiotics and their capacity to regain microbial equilibrium on the skin [55].

Within the realm of eczema, significant research by the National Institute of Allergy and Infectious Diseases (NIAID) resulted in the creation of a topical probiotic derived from *Roseomonas mucosa*, a beneficial skin bacterium. Clinical trials indicated that this probiotic markedly alleviated eczema severity in adults and children, improved skin barrier function, and lowered dependence on corticosteroids. Significantly, the helpful strains remained on the skin for as long as eight months after treatment. This indicates a lasting therapeutic impact and reinforces the importance of microbiome restoration in treating atopic dermatitis [56].

Concerning wound healing, probiotics have demonstrated potential in enhancing tissue repair and minimizing infection. A review in 2024 by Bădăluță et al. highlighted the anti-pathogenic, antibiofilm, and immunomodulatory properties of probiotics, especially in formulations contained in wound dressings. These probiotics assist in managing inflammation, support fibroblast movement, and improve epithelial barrier function [57]. In addition to this, a 2025 study by Zhang et al. showed that the probiotic blend BioK influences crucial signaling pathways, PI3K and TGF-β/Smad, thus facilitating fibroblast migration and decreasing fibrosis. The production of lactic acid by BioK was observed to decrease pH and prevent the differentiation of myofibroblasts, aiding in scar reduction [58].

### 3.5. Oral Microbiome and Probiotic Interventions in Dental and Periodontal Health

The oral cavity hosts one of the most complex and diverse microbial ecosystems in the human body, comprising distinct microbial communities that colonize the teeth, tongue, gingival crevices, and saliva. In a healthy state, the oral microbiome exists in dynamic equilibrium with the host, contributing to immune homeostasis and epithelial barrier integrity. Disruption of this balance, referred to as oral dysbiosis, is closely associated with prevalent oral diseases such as dental caries, halitosis, gingivitis, and periodontitis, and has also been linked to systemic inflammatory and metabolic conditions [59,60,61,62].

Probiotics have gained increasing attention as microbiome-modulating agents capable of restoring oral microbial balance without the broad antimicrobial effects of conventional therapies. Their beneficial effects are mediated through multiple mechanisms, including competitive exclusion of pathogenic species, production of antimicrobial metabolites (e.g., bacteriocins and organic acids), inhibition of pathogenic biofilm formation, and modulation of local immune responses. Probiotic strains belonging primarily to the *Lactobacillus*, *Bifidobacterium*, and *Streptococcus* genera have been most extensively studied for oral health applications [60,61].

In the context of dental caries, probiotics have demonstrated the ability to suppress cariogenic bacteria, particularly *Streptococcus mutans*, by reducing acidogenic activity, competing for adhesion sites on tooth surfaces, and altering biofilm composition. Experimental and clinical studies indicate that probiotic strains such as *Lactobacillus plantarum*, *L. rhamnosus*, and *L. salivarius* can significantly reduce *S. mutans* levels and slow caries development when administered via lozenges, dairy products, or oral hygiene formulations [63].

Halitosis, primarily caused by volatile sulfur compounds produced by anaerobic oral bacteria, has also been shown to respond favorably to probiotic interventions. A randomized, placebo-controlled clinical trial reported that supplementation with *Lactobacillus gasseri* and *L. paracasei* significantly reduced halitosis severity by lowering volatile sulfur compound concentrations, while also exerting systemic metabolic effects, highlighting the interconnected nature of oral and systemic health [8].

In periodontal disease, probiotics have emerged as promising adjuncts to conventional mechanical and non-surgical periodontal therapies. Periodontitis is characterized by chronic inflammation, pathogenic biofilms, and progressive destruction of periodontal tissues. Clinical studies have shown that probiotic supplementation, particularly with *Lactobacillus reuteri*, can reduce periodontal pocket depth, improve clinical attachment levels, and decrease inflammatory markers when combined with scaling and root planning [64]. These effects are attributed to suppression of periodontopathogens such as *Porphyromonas gingivalis*, modulation of host immune responses, and reinforcement of epithelial barrier function.

Overall, current evidence supports the role of probiotics as safe and biologically rational modulators of the oral microbiome. However, their clinical efficacy remains highly strain-specific and dependent on formulation, dosage, and delivery method. While probiotics should not replace established oral hygiene practices or standard dental treatments, their integration into preventive and adjunctive oral healthcare strategies represents a promising microbiome-centered approach.

### 3.6. Metabolic Health

Recent clinical studies have emphasized the potential benefits of probiotics in enhancing metabolic health, especially in the treatment of obesity and type 2 diabetes mellitus (T2DM) [65,66,67]. A network meta-analysis conducted by Allam et al. (2025) aggregated data from 62 randomized controlled trials, revealing that particular probiotic combinations, like those containing *Bifidobacterium bifidum*, *Lactobacillus acidophilus*, and *Lactococcus lactis*, substantially lowered fasting plasma glucose and HbA1c levels in individuals with T2DM [68].

Furthermore, yogurt-based probiotics with *L. acidophilus* La5, *Bifidobacterium* Bb12, and *Cucurbita ficifolia* demonstrated notable effectiveness in managing glycemic levels [68]. Supporting these findings, Maqsood et al. (2025) showed that probiotics such as *Lactobacillus* spp. and *Akkermansia muciniphila* strengthen gut barrier integrity and influence essential metabolic pathways like AMPK and PPAR-γ, thus decreasing inflammation and enhancing insulin sensitivity [69]. Additionally, a bibliometric study conducted by Li et al. (2025) showed an increase in clinical trials centered on probiotics for metabolic disorders, pinpointing obesity, insulin resistance, and hyperlipidemia as significant research focal points [70].

### 3.7. Impact on Obesity, Diabetes, and Lipid Metabolism

Recent clinical studies have shown the positive effects of probiotics on obesity, diabetes, and lipid metabolism, emphasizing their promise as supplementary treatments for metabolic disorders [69,71]. In a 2024 study, Sadeghi et al. assessed 18 meta-analyses and discovered that probiotic supplementation, especially with strains such as *Lactobacillus rhamnosus*, *L. gasseri*, and *Bifidobacterium breve*, resulted in notable decreases in body mass index (−0.30 kg/m^2^), body fat mass (−0.86 kg), and total body weight (−0.59 kg), indicating anti-obesogenic effects via modulation of gut microbiota and inflammation [72]. In addition, Allam et al. (2025) performed a network meta-analysis of 62 randomized controlled trials involving patients with type 2 diabetes mellitus (T2DM), indicating that multi-strain probiotic formulations, such as *Bifidobacterium bifidum*, *Lactobacillus acidophilus*, and *Lactococcus lactis*, markedly enhanced glycemic control and lipid profiles, with significant decreases in fasting plasma glucose and HbA1c [68]. In the field of lipid metabolism, Wang et al. (2023) showed that a 3-month treatment with the combined probiotic formulation Probio-X in hyperlipidemic patients led to reduced serum levels of total cholesterol, triglycerides, and LDL-C, while also raising HDL-C [73]. These impacts were paired with positive changes in gut microbiota composition and lifestyle behaviors, highlighting the diverse advantages of probiotics in addressing dyslipidemia [73].

## 4. Mechanisms of Action

**Figure 2:**
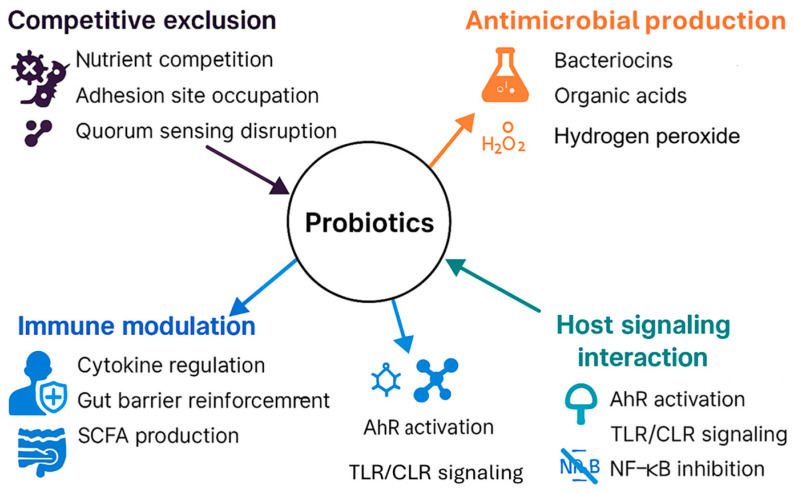
Comprehensive overview of the mechanisms through which probiotics exert their beneficial effects, including competitive exclusion, antimicrobial production, immune modulation, and host signaling interactions.

Probiotics exert their health-promoting effects through several interconnected mechanisms. They competitively exclude pathogens by disrupting quorum sensing and enhancing adhesion to host tissues, thereby preventing colonization and infection [25,74]. Additionally, probiotics produce antimicrobial substances such as bacteriocins and organic acids, which inhibit multidrug-resistant organisms and reduce reliance on conventional antibiotics [15,75]. They also modulate host immune responses by influencing cytokine production, gut barrier integrity, and immune cell differentiation, often mediated through microRNA regulation and SCFA production [76,77,78,79]. Finally, probiotics interact with host signaling pathways via receptors such as AhR, TLRs, and CLRs, influencing key cascades like NF-κB and MAPK to promote immune tolerance and reduce inflammation [80]. Collectively, these mechanisms highlight the therapeutic potential of probiotics in maintaining microbial balance and enhancing systemic health (Figure 2).

### 4.1. Competitive Exclusion of Pathogens

Through a variety of strategies, including competition for nutrients and adhesion sites, as well as interference with microbial communication, probiotics effectively prevent pathogen colonization. This multifaceted mechanism has been increasingly supported by recent research. For instance, Vinayamohan et al. (2024) demonstrated that probiotics can disrupt quorum sensing pathways, which are essential for bacterial virulence, thereby reducing infection severity across the gastrointestinal, pulmonary, and urogenital systems [25]. Building on this, Prajapati et al. (2025) emphasized the role of probiotics in restoring microbial equilibrium, particularly in environments compromised by antibiotic-resistant pathogens, where competitive exclusion becomes critical for preventing dysbiosis [49]. Complementing these findings, Carolak et al. (2025) explored the use of genetically engineered probiotic strains that enhance adhesion to host tissues via surface proteins such as InlA and FnBPA [74]. This targeted adhesion not only strengthens colonization by beneficial microbes but also reinforces their ability to outcompete and exclude pathogenic species [74]. Collectively, these studies underscore the dynamic and evolving understanding of how probiotics assert dominance within microbial ecosystems to safeguard host health.

### 4.2. Production of Antimicrobial Substances

Probiotics generate a diverse range of antimicrobial substances, such as bacteriocins, organic acids, and hydrogen peroxide, which contribute to their ability to suppress pathogenic microbes. Recent studies have expanded our understanding of how these compounds function in clinical and ecological contexts. Thuy et al. (2024) identified bacteriocin-like inhibitory substances (BLISs) from *Lactiplantibacillus plantarum* and *Weissella confusa*, exhibiting strong efficacy against multidrug-resistant pathogens like MRSA and *Pseudomonas aeruginosa* [81]. Extending this line of inquiry, Sarita et al. (2025) examined encapsulation methods that boost the stability and targeted release of antimicrobial metabolites, thereby enhancing their therapeutic effectiveness in complex environments [15]. In a complementary approach, Ezeanya-Bakpa et al. (2024) investigated how probiotics can reduce reliance on conventional antibiotics by directly suppressing pathogen proliferation, thus contributing to the mitigation of antimicrobial resistance [75]. Together, these studies illustrate the diverse biochemical arsenal of probiotics and their growing relevance in combating infectious diseases and antibiotic resistance.

### 4.3. Modulation of Host Immune Responses

Probiotics affect both innate and adaptive immunity by regulating cytokine production, strengthening mucosal barriers, and altering immune cell functions. Recent research has revealed multiple molecular and cellular pathways through which these effects are mediated. Li et al. (2025) demonstrated that probiotics influence host microRNAs (miRNAs), which subsequently impact immune cell differentiation and cytokine synthesis, thereby providing defense against inflammatory bowel disease (IBD) and colorectal cancer [76]. Expanding on this immunological perspective, Mousa et al. (2023) showed that probiotics can reshape gut microbiota composition, modulate antibiotic resistance, and enhance immune tolerance, suggesting a broader role in maintaining immune homeostasis [77].

In the context of autoimmune disorders, Thoda and Touraki (2023) emphasized the importance of short-chain fatty acids (SCFAs) and bacteriocins derived from probiotics in reducing inflammation and reinstating microbial diversity, which are critical for restoring immune balance [78]. Mechanistically, SCFAs (particularly acetate, propionate, and butyrate) can promote regulatory immune programs by signaling through host G-protein-coupled receptors and by inhibiting histone deacetylases, thereby supporting regulatory T-cell polarization and suppressing excessive pro-inflammatory cytokine production. In parallel, bacteriocins contribute indirectly to immune homeostasis by selectively inhibiting pathobionts and reducing pathogen-associated inflammatory stimulation, which helps restore community diversity and lowers the antigenic/inflammatory burden that perpetuates chronic immune activation [78]. Providing a comprehensive overview, Ashaolu et al. (2025) summarized the immunomodulatory actions of probiotics, including gut barrier reinforcement and systemic immune regulation, highlighting their potential as adjuncts in immunotherapy [79]. These studies illustrate the diverse immunological pathways through which probiotics contribute to host defense and immune resilience.

### 4.4. Interaction with Host Signaling Pathways

Probiotics engage with host signaling pathways to affect overall health, especially via receptors such as AhR (aryl hydrocarbon receptor), TLRs (Toll-like receptors), and CLRs (C-type lectin receptors). Recent studies have illuminated the molecular mechanisms through which these interactions occur. De la Rosa González et al. (2024) examined how probiotics stimulate AhR through tryptophan-derived metabolites, enhancing intestinal immune tolerance and lowering inflammatory responses [82]. Building on this receptor-mediated framework, Lee et al. (2023) investigated probiotic effector molecules, including peptidoglycans and extracellular vesicles (EVs), which interact with TLRs and CLRs, thereby influencing key signaling cascades such as NF-κB and MAPK [83]. Extending the role of EVs, Zhang et al. (2025) summarized the function of probiotic-derived extracellular vesicles (PEVs) in microbiota–host communication, emphasizing their promise as postbiotics with systemic signaling capabilities [84]. In a disease-specific context, Hsu et al. (2024) reported evidence indicating that probiotics can stabilize IκBα and inhibit NF-κB activation, leading to a reduction in pro-inflammatory cytokine production in ulcerative colitis [80]. These findings highlight the intricate ways in which probiotics modulate host signaling networks to promote immune balance and systemic health.

## 5. Novel Probiotic Sources, Delivery Systems, and Engineering Strategies

### 5.1. Fermented Foods vs. Pharmaceutical Formulations

Probiotics can be found as food, for example, in fermented foods, drinks, dairy products, and baked goods, or as a drug, which comes in various formulations like powders, capsules, sprays, and liquids [85,86]. Probiotics comprise bacterial cultures including *Lactobacillus*, *Bacillus*, and *Bifidobacterium,* as well as some yeast strains [87]. Milk and dairy products are highly nutritious foods that promote health; however, in lactose-intolerant individuals, fermented dairy products that contain probiotics can be used [88]. The fermentation process is usually supported by the action of *lactobacilli*, which results in augmented nutritional value and enhanced digestibility [89]. Lactic acid fermentation is used for dairy products like cheese, yogurt, and kefir and for vegetables like kimchi [90]. Numerous concerns arise from the use of dairy-based probiotics like lactose intolerance, allergies, and cholesterol levels, rendering non-dairy probiotic products a better alternative [91].

Nutraceuticals are bioactive compounds that are used to augment health and to treat and prevent many diseases [92]. Due to the nature of the probiotics, there are many difficulties concerning their transport to the target site; thus, successful delivery systems should ensure the viability and quantity of the probiotics delivered to gain their benefits [93]. Factors that can affect viability and subsequently the effectiveness of probiotics are thermal stress, oxygen toxicity, gastric low pH, digestive enzymes, and bile salts. These factors can be overcome by either adjusting the method of preparation or the formula [94].

### 5.2. Encapsulation Technologies

Encapsulation techniques present a method to enhance the viability and stability of the probiotics during their delivery to the intestine [95]. The frequently used encapsulation process is microencapsulation, which is categorized into four procedures: spray-drying, freeze-drying, emulsification, and extrusion [96]. New approaches of probiotic delivery are the use of hydrogels and nanostructured platforms, which show promising improvements; furthermore, the use of prebiotics seems to support the proliferation of favorable bacteria [97].

Li et al. (2019) demonstrated that encapsulating *Lactobacillus plantarum* in cellulose microgels enhanced their viability and stability of the product, and allowed controlled release of the bacteria in the targeted tissue [24]. Mojaveri et al. (2020) used innovative electrospun nanofiber mats loaded with *Bifidobacterium animalis* subspecies *lactis* Bb 12 and prebiotic inulin and showed a high melting point and enhanced viability when tested in simulated gastric and intestinal fluids [98]. Furthermore, Hosseini et al. (2022) loaded polyelectrolyte-coated liposomes with the probiotic *L. rhamnosus*, which had enhanced viability in simulated gastric and intestinal fluids [99].

### 5.3. Genetically Engineered Probiotics

Engineered bacteria have recently emerged as powerful next-generation living therapeutics, offering enhanced precision, functionality, and adaptability across medical, environmental, and industrial applications [100]. Due to the apprehensions of conventional probiotics like the variations in their stability, viability and effectiveness, creating genetically engineered probiotics appears necessary. It has been demonstrated that oral engineered probiotics showed better stability, reduced delivery cost, targeted delivery and improved shelf life [101]. Genetic engineering can be used to strengthen an existing probiotic strain to enhance its properties or create an entirely new probiotic [102]. Genetic engineering can be achieved using different methods; however, using it in humans would require deleting the antibiotic resistance genes and transforming the probiotics using their own DNA [103]. The genetic engineering technologies used are homologous recombination, Zincfinger Nucleases (ZFNs), Transcription Activator-Like Effector Nucleases (TALENs) and Clustered Regulatory Interspaced Short Palindromic Repeats (CRISPR)/CRISPR-associated proteins (Cas) [104]. Many studies have demonstrated the benefits of genetically engineered probiotics in various diseases including inflammatory diseases like irritable bowel disease (IBD) and multiple sclerosis and cardio-metabolic disorders [105,106]. In a systematic review, the effectiveness of genetically modulated (GM) probiotics in IBD models was studied. This review indicated that GM probiotics vary in their effectiveness due to variations in the combination used, the wild type used, dose, and the IBD model studied [107]. Furthermore, GM probiotics showed effects through several mechanisms like strengthening the intestinal barrier, releasing numerous modulatory substances, and modulating the ratio between pro- and anti-inflammatory cytokines, the microbiota, and oxidative stress [107].

Studies have demonstrated the utilization of genetically engineered probiotics in developing novel cancer therapy approaches. Chen et al. (2024) showed that genetically engineered *Escherichia coli* Nissle 1917 (EcN) had superb photothermal performance upon near-infrared (NIR) laser irradiation, resulting in vast immunogenic death of cancer cells; furthermore, it promoted the maturation of dendritic cells [108]. Moreover, Ji et al. (2023) demonstrated that genetically engineered *E. coli* MG 1655 could target the tumor cells, specifically triggering catalytic nutrient deprivation followed by autophagy and activation of the p53 apoptosis pathway [109].

Table 4 provides a comparative overview of the main categories of probiotic sources and delivery systems, highlighting their key features, examples, advantages, and associated challenges (Table 4).

## 6. Challenges and Controversies

### 6.1. Regulatory Issues and Labeling

It is important to have clear and thorough regulations and accurate labeling when using a product like probiotics which is intended to be consumed by humans to ensure safety, quality, and efficacy [111]. There have been concerns about the inconsistencies of rules across the globe where standardized regulations should be circulated worldwide [112]; moreover, inaccurate labeling of probiotic products poses substantial apprehension [113]. Fredua-Agyeman and Larbi have analyzed the consistencies between the labels and the factual composition and recommendations of probiotics used in food supplements and food products. This study revealed that there were major inconsistencies between the actual labels and the recommended ones, including non-existent bacteria, excluding existing strains in the product, missing cell concentration information, and the absence of scientific evidence on their health benefits [114]. These discrepancies and lack of proper regulations would trigger significant safety and efficacy concerns, predominantly when probiotics are used clinically to manage major health illnesses [115]. In addition to establishing appropriate regulations for the use and manufacturing of probiotics, perfecting manufacturing formulations and quality control standards and using multi-omics approaches like probiogenomics are suggestions to augment safety, efficacy, and clinical applications [116].

### 6.2. Strain Specificity and Reproducibility

Quality control of commercial probiotic products is important to ensure their quality through ensuring the strain specificity, beneficial doses, and being pathogen-free, as these are intended for human consumption [117,118]. Methods used to test these products’ quality can be enumerating the viable bacteria using plate counting and identification of the bacteria present using Matrix-Associated Laser Desorption/Ionization Time-of-Flight (MALDI-TOF) mass spectrometry [119]. Mora et al. (2019) tested the probiotics taxonomy and identification using metagenomics, their viability using flow cytometry, and the reproducibility of the manufacturing process using β-galactosidase and urase activity [120]. Moreover, metaproteomic analysis could identify the functionality, composition, and reproducibility of the probiotics’ products [120]. Weitzel et al. (2021) proposed a tool named Analytical Procedure Lifecycle Management (APLM) that identifies procedure operation [121]. This tool would decrease the irregularities of using colony-forming units (CFUs) to enumerate probiotics and offer insights on procedural adjustments that would enhance quality control and guarantee clinically beneficial doses [121].

### 6.3. Safety Concerns Regarding Susceptible Individuals

Although probiotics are labeled as Generally Recognized as Safe (GRAS) by the American Food and Drug Administration (FDA) [22], there are concerns about their safety in certain individuals including premature infants, pregnant, immunocompromised individuals, catheterized patients, and people with critical clinical conditions like diabetes mellitus [122,123]. Furthermore, risks of bacteremia, fungemia [122], pneumonia [124], and endocarditis [125,126] in susceptible hosts have been reported.

In a case report, a susceptible elderly patient with several comorbidities had a *Lactobacillus* bacteremia triggered by bacterial transmigration of pathogens due to probiotic use [127]. Similarly, an elderly, immunocompromised patient with various comorbidities like heart failure, kidney disease, and hypertension had been diagnosed with *Lactobacillus rhamnosus* bacteremia that was resistant to antibiotics due to the use of probiotics, which caused his death [128]. These cases highlight the risks and precautions that should be considered when using probiotics, especially over-the-counter products or food products in susceptible individuals.

To ensure that the probiotics used are safe, various bacterial properties should be considered, like gene transfer, produced metabolites, translocation, and immunomodulation effects [123].

Hradicka et al. (2023) studied the effects of long-term supplementation with probiotics in a rat model, where it led to an elevation in pro-inflammatory cytokines, changed the intestinal normal flora population by increasing species associated with inflammation, and elevated levels of cardiovascular risk indicators like the lipoprotein ratio [129]. However, short-term use stimulated the innate immune system and the proliferation of gut protector bacterial species, and these benefits seem to vary depending on the mixture used in the probiotic product [129].

This highlights the variations in the outcomes of probiotic use, where the duration of use can result in contrasting outcomes.

### 6.4. Lack of Standardization in Clinical Trials

Parigi et al. (2023) demonstrated in an international survey that physicians are generally reluctant to prescribe probiotics for IBD cases due to the absence of evidence-based effectiveness and lack of standardization; nevertheless, in cases related to diarrhea and post-antibiotic dysbiosis, prescribing probiotics was more prevalent [130]. Merenstein et al. (2023) recommended that clinical trials on probiotics must declare all adverse effects reported by participants and they should be recorded and defined, with recommendations for standardized conditions if applicable [131].

Risks of adverse effects, measurement of recurrent events, and the number of participants retreated due to adverse effects should all be reported [131]. Various meta-analysis studies indicated that there are variations in the probiotic strains used, duration, dosing, methods used to assess the benefits of probiotics, and their drawbacks in different clinical trials [132,133,134].

In conclusion, the gap in our knowledge about probiotics’ effectiveness and safety requires further studies and clinical trials before probiotics become a standard treatment option in many medical conditions related to psychiatry, autism, IBD and other inflammatory diseases.

## 7. Future Directions

### 7.1. Personalized Probiotics and Microbiome-Based Therapies

Microbiome dysbiosis can lead to many health issues and is associated with several diseases; therefore, personalized medicine offers a tailored treatment plan for each patient depending on their case [135]. In a study done on the perspectives of people regarding the use of microbiome-based therapies to manage mental disorders, subjects seemed keen on the idea of trying these therapeutic approaches, despite having concerns [136]. For instance, Fu et al. (2023) used probiotic-based nanoparticles of OASCLR (onion, hyaluronic acid, chitosan, and living *L. rhamnosus*) for targeted treatment in bacterial pneumonia, and they showed the ability to eliminate pathogens, while modulating the microbiota and immune response in the lungs [137]. Artificial intelligence (AI) algorithms can help in predicting individual reactions based on genetics and immune profiles, thus offering personalized probiotic therapies which can improve outcomes and minimize adverse effects [138]. For precision medicine, it was stated that the selection of participants in clinical trials and practices should be based on certain biomarkers for an ideal benefit–risk equilibrium [139].

### 7.2. Synthetic Biology and Designer Probiotics

Bioresorbable electronics is a term describing transient electronic devices designed to dissolve to biocompatible and environmentally friendly byproducts where these devices can be used for environmental and medical purposes [140,141]. Bioresorbable electronics have the advantage of avoiding consecutive operations on the patients as the temporarily implanted device would resorb without any consequences [140]. Using such electronics would require a biobattery that can also degrade in a similar way. Rezaie et al. (2025) created a probiotic-powered biobattery that showed success in generating power using a commercially available probiotic mixture that functioned as an electrogenic biocatalyst [110]. Using probiotics showed a novel way to utilize these bacteria in creating biocompatible batteries that can be incorporated in bioresorbable electronics used for various applications in the medical and environmental fields [110].

### 7.3. Integration with AI and Big Data for Microbiome Analysis

Identification and classification of microbial communities can be achieved by molecular methods like DNA sequencing; however, it has its limitations, like the huge number of taxonomic groups to be observed. Thus, new approaches using machine learning have been studied [142]. The benefits of using machine learning approaches are automated pattern detection and high-dimensional data handling [143]. Various models have been mentioned where they could be used for classification, identifying biomarkers, and gene prediction, where these are correlated with a human microbiome study [143]. Oh and Zhang presented DeepMicro, a framework for disease prediction based on the microbiome, which can overcome the issues faced by machine learning approaches where dealing with high-dimensional data can be a challenge [144].

Mallick et al. (2019) developed MelonnPan, a computational framework for predictive metabolomics that infers community-level metabolite profiles from microbiome sequencing data using pretrained machine learning models trained on paired metagenomic and metabolomic datasets [145]. Rather than directly measuring metabolites, MelonnPan predicts functional metabolic output based on microbial gene abundance and is therefore primarily suited as a hypothesis generation tool, despite known limitations [145]. In another study, a machine learning model was created to forecast cancer treatment outcomes based on the intestinal microbiome composition and functions, where the microbiome seemed to have a strong relation with cancer growth and prognosis [146]. Validation of the different machine learning models used is crucial; thus, Pasolli et al. (2016) developed a software framework and consistently handled microbiome profiles of samples to assist follow-up research and the evaluation of new approaches [147].

## 8. Conclusions

Probiotics have evolved from their traditional function in gut health to a complex therapeutic framework affecting overall body physiology. Evidence highlights their ability to regulate immune responses, improve metabolic stability, and aid neurological and skin health via intricate microbiome interactions. Even with encouraging results, obstacles remain concerning strain specificity, regulatory uniformity, and safety for at-risk groups. New technologies, like encapsulation, engineered microorganisms, and AI-based microbiome analysis, provide avenues to tackle these challenges and facilitate precise probiotic treatments. Future studies must emphasize interdisciplinary methods that combine microbiology, immunology, neuroscience, and bioengineering to develop strong clinical evidence and sustainable production frameworks. Ultimately, probiotics serve as a foundation for next-generation healthcare, possessing the ability to revolutionize preventive and therapeutic approaches across various medical fields. By integrating systemic microbiome–host interactions with shared mechanistic pathways and emerging translational strategies, this review provides a systems-level synthesis that moves beyond descriptive summaries and supports the rational design of next-generation probiotic interventions.

## Figures and Tables

**Table 1:** Classical probiotic functions related to gut health and digestive processes, including microbiota balance, antimicrobial activity, barrier reinforcement, and clinical benefits in IBD management.

Function	Mechanism/Effect	Associated Strains	References
**Microbiota Balance**	Maintains gut flora and mucosal integrity	*Lactobacillus*, *Bifidobacterium*	[13,14]
**Antimicrobial Action**	Produces bacteriocins and SCFAs to inhibit pathogens	General strains from *Lactobacillus* and *Bifidobacterium*	[15]
**Digestive Support**	Enhances nutrient absorption, regulates motility, improves lactose digestion	*L. plantarum*, *L. acidophilus*, *L. rhamnosus*	[14]
**Barrier Reinforcement**	Increases tight junction proteins, reduces zonulin levels	Multiple strains from *Lactobacillus* and *Bifidobacterium*	[16]
**Inflammation Control**	Reduces CRP and TNF-α levels	Multiple strains from *Lactobacillus* and *Bifidobacterium*	[16]
**IBD Management**	Reduces disease activity, promotes remission	VSL#3, *E. coli* Nissle 1917	[21]

**Table 2:** Immunomodulatory roles of probiotics highlighting their interactions with innate and adaptive immune cells, cytokine regulation, and modulation of key inflammatory signaling pathways.

Function	Mechanism/Effect	Associated Strains	References
**Immune Cell Interaction**	Modulates dendritic cells, macrophages, lymphocytes	*Lactobacillus*, *Bifidobacterium*	[22]
**Treg Activation**	Promotes IL-10 and TGF-β production	*Lactobacillus*, *Bifidobacterium*	[22]
**Mucosal Immunity**	Increases secretory IgA production	*Lactobacillus*, *Bifidobacterium*	[16]
**Inflammation Reduction**	Lowers CRP, IL-6, TNF-α	*Lactobacillus*, *Bifidobacterium*	[16,23]
**Immune Homeostasis**	Rebalances immune responses in dysbiosis and autoimmune conditions	General strains	[23]
**Signal Pathway Modulation**	Influences NF-κB and MAPK pathways	*L. acidophilus*, *B. animalis* subsp. *lactis*	[24]

**Table 3:** Probiotic-mediated mechanisms in the prevention of gastrointestinal infections through pathogen exclusion, barrier enhancement, antimicrobial compound production, and reduction in antibiotic-associated diarrhea.

Function	Mechanism/Effect	Associated Strains	References
**Pathogen Exclusion**	Competes for adhesion sites and nutrients	*Lactobacillus*, *Bifidobacterium*	[25]
**Barrier Enhancement**	Strengthens mucosal defenses and gut integrity	Multi-strain formulations	[26]
**Antimicrobial Production**	Secretes organic acids, hydrogen peroxide, bacteriocins	*Bacillus* spp., *Lactobacillus* spp.	[28]
**Antibiotic-Associated Diarrhea**	Reduces incidence by 37%	*Lactobacillus*, *Bifidobacterium*	[27]
**Drug-Resistant Pathogen Defense**	Produces targeted metabolites, supports immunity	*Bacillus* spp.	[29]
**Pathogen Inhibition**	Suppresses *E. coli*, *Shigella*, *C. difficile*	*L. rhamnosus*, *B. lactis*, *S. boulardii*	[30]

**Table 4:** Overview of novel probiotic sources, delivery technologies, and formulation strategies, including fermented foods, pharmaceutical preparations, encapsulation approaches, genetically engineered strains, and advanced delivery platforms.

Category	Key Features	Examples/Techniques	Advantages	Challenges	References
**Fermented Foods**	Natural dietary sources of probiotics	Yogurt, kefir, kimchi, sauerkraut	Affordable, accessible, culturally accepted	Lactose intolerance, allergies, strain variability	[86,90,91]
**Pharmaceutical Formulations**	Controlled dosing	Capsules, powders, sprays, liquids	Precise dosing, longer shelf life	Stability loss during storage and GI transit	[85,86]
**Encapsulation Technologies**	Protection during GI transit	Spray-drying, freeze-drying, emulsification, extrusion; hydrogels; nanofibers	Enhanced viability and controlled release	Expensive and complex manufacturing	[95,97,98,99]
**Prebiotic Integration**	Probiotic + prebiotic synergy	Inulin, FOS	Enhances growth of beneficial bacteria	Requires precise formulation	[62,94]
**Genetically Engineered Probiotics**	Modified strains for improved function	CRISPR, TALEN, ZFNs, homologous recombination	Targeted delivery and enhanced activity	Ethical and regulatory concerns	[101,104,107]
**Novel Delivery Platforms**	Innovative carriers	Polyelectrolyte-coated liposomes; cellulose microgels; bioresorbable electronics	Controlled, site-specific delivery	High cost; safety validation needed	[99,110]

## Data Availability

No new data were created or analyzed in this study. Data sharing is not applicable.
